# Shear bond strength of acid and laser conditioned enamel and dentine to composite resin restorations: An in vitro study

**DOI:** 10.1002/cre2.409

**Published:** 2021-02-18

**Authors:** Al Hanouf Al Habdan, Reem Al Rabiah, Riham Al Busayes

**Affiliations:** ^1^ Department of Restorative Dental Sciences King Saud University Riyadh Saudi Arabia; ^2^ College of Dentistry King Saud University Riyadh Saudi Arabia

**Keywords:** composite resin, dentin, enamel, laser

## Abstract

**Objective:**

To compare the shear bond strength of enamel or dentin conditioned with either Er,Cr:YSGG (erbium, chromium: yttrium–scandium–gallium–garnet) laser or phosphoric acid to composite resin restoration.

**Material and methods:**

Forty posterior human extracted teeth were used. After mesiodistal sectioning of the teeth crowns, the samples were randomly divided into two groups—in the first group (E), bonding was performed on the enamel after roughening and in the second group (D), the enamel was removed and bonding was performed on the dentin. These groups were further randomly divided into two subgroups according to the type of etching (*n* = 20 each). In the acid‐etched groups (EA and DA), the surfaces were etched with 37% phosphoric acid. In the laser‐conditioned groups (EL and DL), the surfaces were conditioned with Er,Cr:YSGG laser. Total‐etch adhesive system was used to bond all the 80 specimens resin composite. The composite was vertically light‐cured, and the specimens were subjected to a shear bond strength test. Modes of bond failure were determined with a stereomicroscope.

**Results:**

The highest shear bond strength was observed for the DA group (16.25 ± 1.10 MPa, *p* < 0.0001), whereas the lowest was observed for the DL group (8.56 ± 0.67 MPa). The adhesive failure mode was the most frequently observed in all groups.

**Conclusions:**

The shear bond strength of composite resin bonded to enamel and dentin etched with phosphoric acid was higher than when conditioned with Er,Cr:YSGG laser. Thus, laser conditioning is not recommended.

## 1 INTRODUCTION

In 1955, Buonocore introduced acid etching that resulted in a very significant increase in adhesion on the tooth–restoration interface. Acid etching removes the smear layer generated by cavity preparation. This layer is characterized by a low surface energy that reduces the strength of the bond between biomaterials and enamel or dentin (Buonocore, 1955). This bonding mechanism involves micromechanical interlocking of resin tags into the dentinal pores created by acid etching (De Munck et al., 2005).

Composite resin restorations are commonly used to restore dental structures, but they typically illustrate lower bond strength when used on dentin compared with enamel (De Munck et al., 2005). Progressing to increase the strength of this bond, several adhesive systems have been introduced (Nasseri, Majidinia, & Sharbaf, 2017). Per the technique used and also the mechanism of adhesion, adhesive systems are broadly categorized into two main categories: total‐etch and self‐etch adhesive systems (Gupta et al., 2017). Many companies produce total‐etch adhesive systems as either a three‐step system (acid etchant, primer, and adhesive) or a two‐step system (acid etchant, and a combination of primer and adhesive in a single bottle) (Rechmann, Bartolome, Kinsel, Vaderhobli, & Rechmann, 2017). Self‐etch adhesive systems are composed of a self‐etching primer and an adhesive resin that's either provided in two separate bottles (two‐step system) or combined in a single bottle (one‐step system). Three‐step total‐etch adhesives are believed to be the gold standard in enamel bonding thanks to the effective bond formed after the utilization of the solvent‐free, neutral pH, hydrophobic, and adhesive resin layer as a separate step (Raposo & Santana, 2012).

Several studies have investigated the likelihood of replacing the use of acid with newer techniques such as laser etching (Nelson, Wefel, Jongebloed, & Featherstone, 1987). The evolution of lasers in dentistry has facilitated the development of various soft and hard tissue procedures including soft tissue surgeries, dental bleaching, restorative curing, and painless caries removal and tooth preparation (Turkmen et al., 2010). Several kinds of lasers are utilized in dental practice such as the Nd:YAG laser, which is not well‐absorbed by hard dental tissues, and the carbon dioxide laser, which might cause an a rise in pulpal temperature (van As, 2004). These limitations have been eliminated by the introduction of the erbium (Er) family of lasers, which were approved in 1998 by the US FDA for irradiating tooth surfaces (Ustunkol, Yazici, Gorucu, & Dayangac, 2015).

There are two known wavelengths of Er lasers in the dental field: Er,Cr:YSGG (Er, chromium: yttrium–scandium–gallium–garnet) lasers (~2780 nm) and Er:YAG (Er: yttrium–aluminum–garnet) lasers (~2940 nm). These wavelengths show *high* absorbability by both water and hydroxyapatite compared with any other dental laser wavelengths. Therefore, Er lasers are considered optimal for to be used on hard dental tissues. Successful dental ablation can be achieved with Er,Cr:YSGG laser because of its shorter wavelength, high absorption by water and enamel, and also the laser's water‐cooled system allows control of the pulpal temperature (Kumar, Dhillon, & Rehman, 2016). Several studies have also proven that enamel and dentin surfaces conditioned with Er,Cr:YSGG laser lead to the removal of the smear layer and formation of micro‐irregularities along the dental surface (Ustunkol et al., 2015). Laser conditioning alters the calcium/phosphorus ratio on the dental surface, and these changes provide the enamel with resistance against caries attacks (Kumar et al., 2016).

The use of lasers for enamel conditioning is controversial, as some investigations have shown that lasers do not seem to be always fully effective for this purpose. Despite their advantages of being heatless and painless, laser were found by some investigators to create uneven enamel surface with a lot of fracture areas (Usumez & Aykent, 2003; von Fraunhofer, Allen, & Orbell, 1993). Usumez et al. in 2002 stated that “enamel conditioning with an Er,Cr:YSGG laser cannot be considered a successful alternative to the conventional methods of increasing bond strengths to enamel” (Usumez, Orhan, & Usumez, 2002). In contradictory, other researchers have reported satisfactory results and increased bonding strength between the enamel and composite resin after laser conditioning (Basaran, Ayna, Basaran, & Beydemir, 2011; Hossain et al., 2003; Visuri, Gilbert, Wright, Wigdor, & Walsh Jr., 1996). They reported that Er,Cr:YSGG laser increase enamel acid resistance by altering calcium to phosphorus ratio and carbonate to phosphorus ratio within enamel structure (Fowler & Kuroda, 1986; Keller & Hibst, 1990). In addition, it was found that laser ablation with a power of 2 W (5.6 J/cm^2^) produces an etch pattern that resembles type III acid etching pattern with surface roughness similar or less to that produced by conventional acid etching (Silverstone, Saxton, Dogon, & Fejerskov, 1975). There's also debate concerning the subject of dentin bonding; many studies have found that the utilization of phosphoric acid for etching dentin before composite resin restorations yields increased shear bond strength in comparison with laser conditioning (Armengol, Jean, Weiss, & Hamel, 1999; Dunn, Davis, & Bush, 2005; Jaberi Ansari et al., 2012). On the other hand, other studies reported that equal or higher shear bond strength after laser conditioning of dentin (Bertrand et al., 2006; Visuri et al., 1996). Therefore, this study was aimed to evaluate the shear bond strength enamel or dentin conditioned with either Er,Cr:YSGG (erbium, chromium: yttrium–scandium–gallium–garnet) laser or phosphoric acid to composite resin restorations.

## 2 MATERIALS AND METHODS

### 2.1 Specimen preparation

In this study, posterior human extracted teeth were used. The teeth were thoroughly inspected for caries, cracks, fluorosis, abrasion facets, and damage from extraction and 40 teeth were selected. Samples were thoroughly washed and stored in dark glass containers in 1% (v/v) thymol solution at 4°C after extraction and used within 2 months. IsoMet 2000 Precision Saw (Buehler, Ltd., Lake Bluff, IL, USA) used to cut the roots below the furcation. Mesiodistal sectioning of the teeth crowns was performed, and both buccal and lingual surfaces were used for this study. Sectioned samples were mounted in acrylic resin mold where the sectioned surface was positioned facing the resin. The samples were then divided randomly into two groups with half of the sample surfaces being subjected to enamel roughening with 600‐grit disk (Automata Grinding and Polishing Unit; Jean Wirtz GmbH, Dusseldorf, Germany) and continuous air–water irrigation to resemble simple cavity preparation in enamel. The other half of the samples were subjected to different grits of abrasive disks (320‐grit/400‐grit/and 600‐grit) to get rid of the enamel layer and expose the dentin (Automata Grinding and Polishing Unit). The ground samples were then placed in an ultrasonic machine (Sonicer; Yoshida Dental Mfg. Co., Ltd., Osaka, Japan) filled with distilled water for 15 min to remove the enamel debris. To confirm complete enamel removal specimens were examined under a light microscope (Stereo 80 Widefield Microscope; Swift Optical Instruments, Schertz, TX, USA). Finally, the samples were randomly divided into two groups—in the enamel group (E), the bonding was performed on the enamel, whereas in the dentine group (D), the bonding was performed on the dentin. These groups were further divided randomly into two subgroups per the kind of etching used: either phosphoric acid etching (EA and DA) or Er,Cr:YSGG laser conditioning (EL and DL) with 20 samples in each subgroup. All the materials and equipment utilized in this study were applied per the manufacturers' instructions.

### 2.2 Bonding procedure

A customized silicon mold with a thickness of 2 mm was fabricated to be used in the composite bonding procedure. A 3‐mm circular hole was made in the center of this mold. The mold was placed within the center of the enamel/dentin surface. In the acid‐etched groups (EA and DA), the demarcated surfaces were etched with 37% (v/v) phosphoric acid (Total Etch; Ivoclar Vivadent, Schaan, Liechtenstein) for 30 s, washed, and dried. In the laser‐conditioned groups (EL and DL), the demarcated surfaces were etched with Er,Cr:YSGG laser (Waterlase iPlus; Biolase, Irvine, CA, USA) and the laser tip (MZ8, 6 mm in length) was applied perpendicular to the tooth surface and moved slowly along the surface for 30 s with 0.5‐mm distance from the surface in scanning mode. The laser had a wavelength of 2780 nm, power of 4.50 W, 50 Hz frequency in H mode, 60% air, 30% water spray, pulse duration of 140 μs, energy density of 90 mJ/pulse, and 800 nm spot size.

Following the above treatments, all 80 specimens were ready for the application of the total‐etch adhesive system (ExciTE F; Ivoclar Vivadent, Schaan, Liechtenstein) per manufacturer's instructions. Resin composite (Filtek Z250XT Universal Restorative; 3M ESPE, St. Paul, MN, USA) was applied to the enamel and dentin surfaces and packed gently with a plastic instrument. A very thin celluloid strip was placed on top of the composite to stabilize it. The composite was vertically light‐cured for 20 s with a previously calibrated LED light‐curing device (Bluephase G2; Ivoclar Vivadent, Schaan, Liechtenstein) per manufacturer's instructions. The celluloid strip and mold were gently removed, and the samples were cured again for 20 s. All the materials used are described in Table 1.

**TABLE 1:** Description of materials used

Material	Brand name (company)	Composition	
Phosphoric acid etchant	Total Etch (Ivoclar Vivadent, Schaan, Liechtenstein)	Total etch contains phosphoric acid (37% (v/v) in water), thickening agent, and color pigments	
Total Etch adhesive system	ExciTE F (Ivoclar Vivadent, Schaan, Liechtenstein)	Phosphoric acid acrylate, hydroxyethyl methacrylate, methacrylate	(%, g/v) 77.9
		Highly dispersed silica	0.5
		Ethanol	19.5
		Catalysts, stabilizers, fluoride	2.1
Composite resin	Filtek Z250XT Universal Restorative (3M ESPE, St. Paul, MN, USA)	The filler system: Surface‐modified zirconia/silica with a median particle size of ≤3 μm Non‐agglomerated/non‐aggregated 20‐nm surface‐modified silica particles The filler loading was 82% by weight (68% by volume) The resin system: BIS‐GMA, UDMA, BIS‐EMA, PEGDMA, and TEGDMA	

### 2.3 Shear bond strength testing

The specimens were subjected to shear bond strength testing with a universal testing machine (Instron 5965; Instron, England) with a load cell of 5 kN operated by a single operator. A knife‐edged rod with a width of 0.5 mm was applied at the interface of the resin composite disk with the enamel/dentin at a crosshead speed of 0.5 mm/min. Modes of bond failure were determined by viewing the fracture sites along the enamel/dentin–composite disk interface under a stereomicroscope (Nikon Stereomicroscope 100 m Microscope, SMZ 1000, SMZ800, Swift, CA, USA) with a digital camera (Nikon digital camera DXM1200F). Failure modes were classified as adhesive, cohesive, or mixed and were defined as follows: adhesive failure showed no sign of dentin/enamel fracture or remnants of composite resin on the tooth; cohesive fractures showed complete fracture of dentin or resin; and mixed samples showed both adhesive and cohesive failures.

### 2.4 Statistical analysis

One‐way analysis of variance was utilized to compare the mean values of shear bond strength among all four groups, followed by Tukey's test for pairwise comparisons. *p*‐values of <0.05 were considered statistically significant.

### 2.5 Ethical approval

This study has been approved by the Institutional Review Board (IRB Project No. E‐17‐2643), College of Medicine, King Saud University and the College of Dentistry Research Center (CDRC No. IR 0251), King Saud University.

## 3 RESULTS

The descriptive statistics (minimum, maximum, mean, and standard deviation) of the shear bond strength (MPa = N/mm^2^), which was measured at maximum load (N), are given in Table 2.

**TABLE 2:** Descriptive statistics of the shear bond strength of the four study groups

	Groups	No.	Mean	Std. deviation	Minimum	Maximum
Shear bond strength (MPa)	EL	20	11.44	0.75	10.34	12.80
	EA	20	15.36	0.75	14.03	16.84
	DL	20	8.56	0.67	7.28	9.66
	DA	20	16.25	1.10	14.58	18.08

Abbreviations: DA, dentin etched with phosphoric acid; DL, dentin etched with laser; EA, enamel etched with phosphoric acid; EL, enamel etched with laser.

Comparison of the mean shear bond strength among the four study groups (EL, EA, DL, and DA) showed a statistically significant difference (*p* < 0.0001). Tukey's pairwise comparison between each of the four study groups showed that the mean shear bond strength of the DA group was statistically significantly higher than that of the other three groups (DL, EL, and EA), whereas the mean for the DL group was statistically significantly lower than that of the other three groups. The statistical results are summarized in Tables 3 and 4.

**TABLE 3:** Comparison of the shear bond strength among the four study groups (using one‐way analysis of variance)

		Sum of squares	Dff	Mean square	*F*‐value	*p*‐value
Shear bond strength (MPa)	Between groups	764.199	3	254.733	361.949	<0.0001
	Within groups	53.487	76	0.704		
	Total	817.686	79			

**TABLE 4:** Pairwise comparison of shear bond strength between the four study groups (using Tukey's test)

Outcome variable	(*I*) Groups	(*J*) Groups	Mean difference (*I* − *J*)	*p*‐value	95% confidence interval	
					Lower bound	Upper bound
Shear bond strength (MPa)	EL	EA	−3.92^*^	0.0001	−4.61	−3.22
		DL	2.87^*^	0.0001	2.17	3.57
		DA	−4.81^*^	0.0001	−5.50	−4.11
	EA	EL	3.92^*^	0.0001	3.22	4.61
		DL	6.79^*^	0.0001	6.10	7.49
		DA	−0.88^*^	0.007	−1.58	−0.19
	DL	EL	−2.87^*^	0.0001	−3.57	−2.17
		EA	−6.79^*^	0.0001	−7.49	−6.10
		DA	−7.68^*^	0.0001	−8.38	−6.98
	DA	EL	4.81^*^	0.0001	4.11	5.50
		EA	0.88^*^	0.007	0.191	1.58
		DL	7.68^*^	0.0001	6.98	8.38

Abbreviations: DA, dentin etched with phosphoric acid; DL, dentin etched with laser; EA, enamel etched with phosphoric acid; EL, enamel etched with laser.

* Mean difference statistically significant (*p* < 0.05).

**FIGURE 1:**
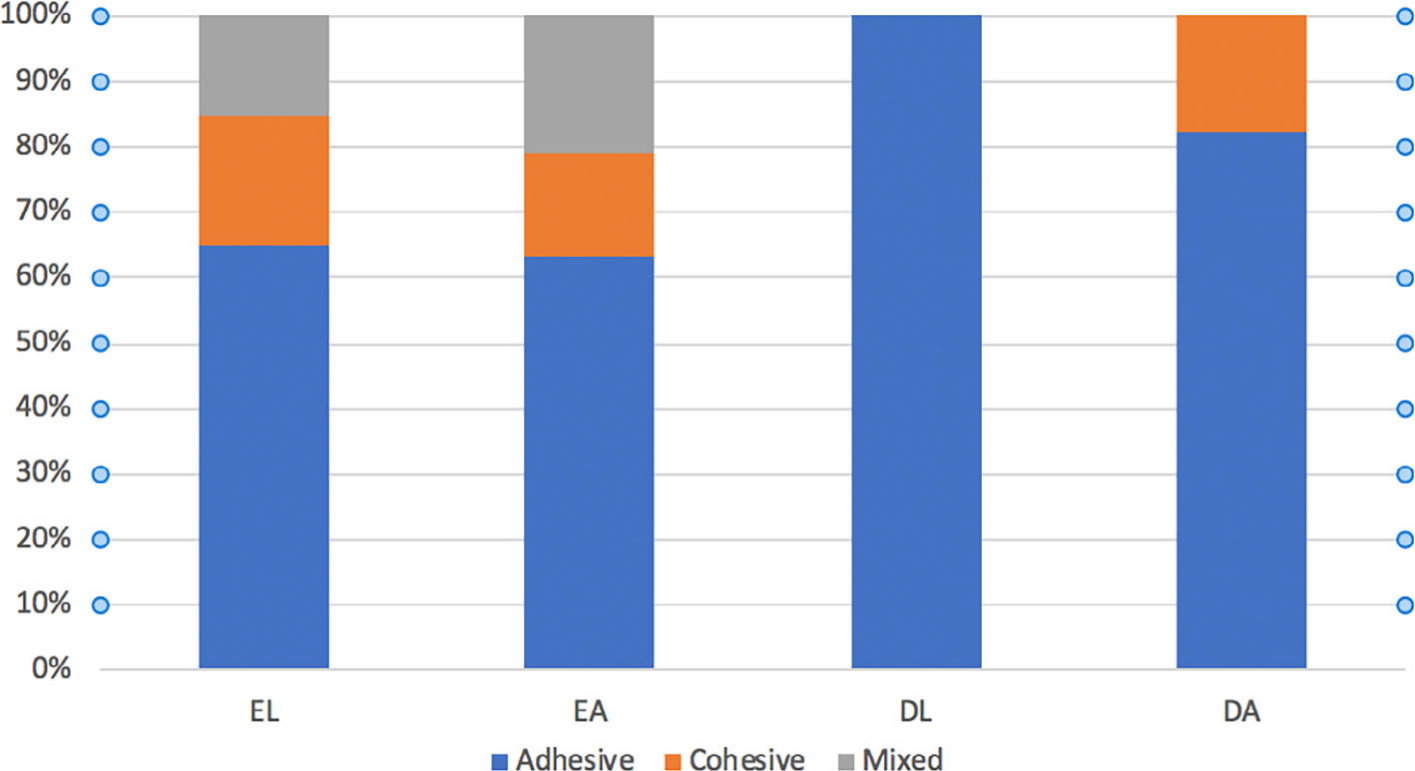
Proportion of failure modes in each of the four study groups. All four study groups of resin composite bonded to enamel or dentin were subjected to shear bond strength testing. DA, dentin etched with phosphoric acid; DL, dentin conditioned with laser; EA, enamel etched with phosphoric acid; EL, enamel conditioned with laser

**FIGURE 2:**
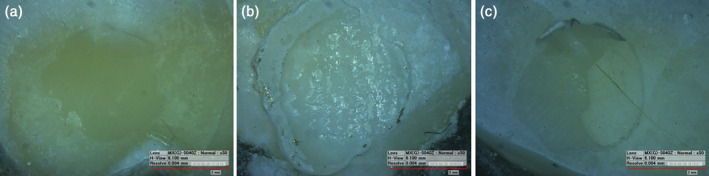
Microscopy of representative specimens after testing the shear bond strength with a universal testing machine. (a) Adhesive failure with exposure of dentin. (b) Cohesive failure with resin residual on the sample. (c) Mixed failure with resin residual on the sample and partial exposure of the tooth structure

The percentages of samples exhibiting the three failure modes in each group are shown in Figure 1. Adhesive failure was the most predominant failure mode for all the tested groups, followed by the cohesive and mixed failure modes that were equally prominent overall across three of the groups (EA, EL, and DA). The adhesive failure mode was the only mode to occur in the DL group. Examples of the different failure modes are displayed in Figure 2a–c.

## 4 DISCUSSION

To date resin composite bond strength to enamel and dentine conditioned with Er,Cr:YSGG lasers remains debating. Laser conditioning of enamel and dentin require different laser parameter than that used for cavity preparation. The laser type utilized in the current study features a fixed parameter by the manufacture for conditioning which is the parameter applied to perform this study (4.5 W/50 Hz). Various features of Erbium lasers have been manipulated and assessed in several studies aiming to control the results and offer higher bond strength and a tighter marginal seal (Basaran, Ayna, et al., 2011; Jaberi Ansari et al., 2012; Usumez et al., 2002). For instance, Usumez and Aykent (2003) and Usumez et al. (2002) adjusted the laser wavelength and irradiated the enamel surface with Er,Cr:YSGG laser at a power output of (2 W, 20 Hz, 100 mJ) or (1 W, 20 Hz, 50 mJ). Decreasing the power to half decreased the bond strength of the irradiated surface, although variable results were recorded. Within the current study, a power of 4.50 W with energy density of 90 mJ was utilized to irradiate the enamel and dentin groups and higher shear bond strength was achieved compared with that obtained in previous studies (Usumez et al., 2002; Usumez & Aykent, 2003).

Many bond strength studies use microtensile or tensile bond strength tests to predict the clinical performance of restorative materials (De Munck, Van Meerbeek, Yudhira, Lambrechts, & Vanherle, 2002; Lee et al., 2007; Trajtenberg, Pereira, & Powers, 2004; Van Meerbeek, De Munck, Mattar, Van Landuyt, & Lambrechts, 2003). However, it was confirmed by some investigators that the major stresses involved in the clinical failure of restorative material were mainly shear stresses (Oilo, 1987; Swift, Perdigão, & Heymann, 1995). Therefore, shear bond strength test were used in this study to evaluate composite restoration bond strength to laser etched and acid etched enamel and dentine.

Several studies have proven that laser conditioning of enamel surfaces is useful (Basaran, Hamamci, & Akkurt, 2011; Hossain et al., 2003; Turkmen et al., 2010); however, others have produced contradictory results (Dunn et al., 2005; Martinez‐Insua, Da Silva Dominguez, Rivera, & Santana‐Penin, 2000; Ramos et al., 2002). Yu, Kimura, Kinoshita, and Matsumoto (2000) stated that enamel structure roughness observed after conditioning with Er,Cr:YSGG laser (6 W, 20 Hz, 300 mJ) increases the bond strength of composite restorations. In another study, it had been observed that although the mean enamel bond strength of the acid‐etched group was higher beyond that of the laser‐conditioned group, this difference wasn't significant (Ustunkol et al., 2015). These results can be explained by the effect of Er,Cr:YSGG laser irradiation on enamel surfaces, which show a chalky surface when viewed with scanning microscope. This surface provides increased retention of composite filling material, which is valuable in the restorative procedure (Hibst, 2002; Hoke, Burkes Jr., Gomes, & Wolbarsht, 1990). On the opposite hand, it had been found that laser irradiation of enamel surfaces causes loss of the unique etching pattern that usually appears after acid etching. This effect prevents resin interlocking into the enamel, consequently lowering enamel bond strength (Dunn et al., 2005). Moreover, Jaberi Ansari et al. (2012) found that bur‐cut and laser etch enamel recorded the lowest shear bond strength values among all enamel group tested in their study. In this study, consistent results were observed as enamel samples etched with 37% phosphoric acid showed higher shear bond strength than those irradiated with Er,Cr:YSGG laser.

In the current study, dentin surfaces etched with phosphoric acid demonstrated the highest shear bond strength among the four groups, while laser‐irradiated dentin surfaces exhibited the lowest shear bond strength. Chou, Chen, and Ding (2009) stated that there's no significant difference in shear bond strength between laser‐conditioned or acid‐etched groups. In addition, a study by Lin, Caputo, Eversole, and Rizoiu (1999) used Er,Cr:YSGG laser at a parameter of 4w and 20 Hz found no significant difference in the shear bond strength between laser etch and acid etch dentine. Moreover, Sung et al. (2006) recorded higher shear bond strength values of dentine etched with Er,Cr:YSGG laser (4–5 W) which is in agreement with a study done by Gurgan et al. (2008). This outcome can be illustrated by the changes noticed in the composition and conformation of the organic matrix that might result in collagen degradation and deterioration of adhesive penetration (Bachmann, Diebolder, Hibst, & Zezell, 2005). Furthermore, Erbium laser irradiation on dentin causes odontoblastic tubules to open up, and dentin shows surface scaling after the application of laser, and this often results in flaking and peritubular cuffing. This odd manifestation of dentin is explained by Lin et al. (1999) as micro‐explosions within the inorganic structures in the teeth that appear after Er,Cr:YSGG laser irradiation. It had been also proposed by Sennou, Lebugle, and Gregoire (1999) that laser conditioning of dentin binds collagen fibrils together, which results in the absence of interfibrillar space and thus prevention of resin penetration into the intertubular dentin happens. This result might explain the low shear bond strength of laser conditioned dentin in the current study.

The failure modes of the samples were also tested during this study, and therefore the most frequent failure mode among all the four study groups was the adhesive failure mode. It is notable that the group with the lowest shear bond strength (DL) was also the group demonstrating only the adhesive failure mode. This results in agreement with a study done by Lee et al. (2007) who found that Er,Cr:YSGG laser irradiation adversely affect dentin adhesion to resin composite because laser irradiation produce scaly, irregular surface with no smear layer and open dentinal tubule.

The diverse and often contradictory results of previous studies could be due to the application of different technical parameters, including the physical parameters of the laser or the kind of restorative material used. Further studies are therefore required to verify the main conclusions of our study.

## 5 CONCLUSIONS

Within the limitation of this in vitro study, it can be concluded that laser conditioning of enamel and dentin is not recommended because:

1. The shear bond strength of enamel and dentin groups etched with phosphoric acid was higher than that of groups ablated with Er,Cr:YSGG laser.
2. Adhesive failure was the foremost predominant failure mode for all the tested groups, and it had been the sole failure mode apparent in a laser‐irradiated dentin group.

## CONFLICT OF INTEREST

The authors declare that they have no conflict of interest.

## AUTHOR CONTRIBUTION

All authors have contributed to study design and consecution, writing, revision, and proofing the manuscript.

## Data Availability

The data that support the findings of this study are available from the corresponding author upon reasonable request.
